# Developments in Methods for Measuring the Intestinal Absorption of Nanoparticle-Bound Drugs

**DOI:** 10.3390/ijms17071171

**Published:** 2016-07-21

**Authors:** Wei Liu, Hao Pan, Caiyun Zhang, Liling Zhao, Ruixia Zhao, Yongtao Zhu, Weisan Pan

**Affiliations:** 1 Department of Pharmaceutics, School of Pharmacy, Shenyang Pharmaceutical University, 103 Wenhua Road, Shenyang 110016, China; liuweiyxy@zzu.edu.cn; 2 Department of Pharmaceutics, School of Pharmacy, Zhengzhou University, 100 Science Avenue, Zhengzhou 450001, China; yuncaizzu@163.com (C.Z.); 18749452032@163.com (L.Z.); rxzhao1012@163.com (R.Z.); zhuxiaotao@163.com (Y.Z.); 3 School of Pharmacy, Queen’s University Belfast, Belfast BT7 1NN, UK; haopan87@hotmail.com

**Keywords:** drug delivery, polymeric nanoparticles (NPs), intestinal absorption, in vitro, in situ, in vivo

## Abstract

With the rapid development of nanotechnology, novel drug delivery systems comprising orally administered nanoparticles (NPs) have been paid increasing attention in recent years. The bioavailability of orally administered drugs has significant influence on drug efficacy and therapeutic dosage, and it is therefore imperative that the intestinal absorption of oral NPs be investigated. This review examines the various literature on the oral absorption of polymeric NPs, and provides an overview of the intestinal absorption models that have been developed for the study of oral nanoparticles. Three major categories of models including a total of eight measurement methods are described in detail (in vitro: dialysis bag, rat gut sac, Ussing chamber, cell culture model; in situ: intestinal perfusion, intestinal loops, intestinal vascular cannulation; in vivo: the blood/urine drug concentration method), and the advantages and disadvantages of each method are contrasted and elucidated. In general, in vitro and in situ methods are relatively convenient but lack accuracy, while the in vivo method is troublesome but can provide a true reflection of drug absorption in vivo. This review summarizes the development of intestinal absorption experiments in recent years and provides a reference for the systematic study of the intestinal absorption of nanoparticle-bound drugs.

## 1. Introduction

Polymeric nanoparticles (NPs) [1] are solid colloidal particles smaller than 1 μm in diameter comprising natural or synthetic polymer materials, and include nanospheres [2] and nanocapsules [3]. Drugs can be embedded in the NPs’ interior or adsorbed on the surface, and NPs therefore have potential for incorporation into a variety of drug delivery systems. A relatively new type of drug carrier, NPs have the advantage of strong drug adhesion, which increases the drug’s contact time with the intestinal mucosa thereby enhancing drug absorption. NP-based delivery can be used to influence drug distribution, control drug release rates, improve oral drug bioavailability and reduce side effects [4]. Therefore, NP-based targeted delivery systems could be used for broad applications in the delivery of DNA, polypeptides, proteins, immunomodulators, antineoplastics and so forth.

Over the last few decades, the application of nanoparticles has been investigated for different routes of drug administration, which has opened up new possibilities for facing the shortcomings of current conventional dosage systems [5]. Although there have been tremendous innovations in different drug delivery systems, the oral route is still the most widespread and popular drug administration method by virtue of its convenience, low cost and high patient compliance compared with alternative routes [6,7,8]. However, the vulnerability of many drugs to the harsh conditions of the gastrointestinal (GI) tract and the possibility of chemical and enzymatic degradation results in poor therapeutic efficacy of many orally administered drugs [9]. In fact, oral administration, like other administration routes, faces major obstacles relating to the effective delivery of therapeutics, with the most severe of these being the possibility of drug degradation, negligible transport of drugs across the intestinal epithelium, decreased efficacy due to pH variations and the presence of digestive enzymes, and cell membrane barriers in the GI tract [10]. Thus, it is important to design appropriate models for predicting and screening the permeability and absorption of nanoparticulate drug delivery systems in the intestine [11,12]. This review aims to provide a critical overview of the use and applications of the various existing intestinal absorption models. At present, the intestinal absorption models of oral NP-based drug delivery systems can be classified into three categories comprising a total of eight different measurement methods as shown in Table 1 [13], namely in vitro (dialysis bag, rat gut sac, Ussing chamber, and cell culture model), in situ (intestinal perfusion, intestinal loops, and intestinal vascular cannulation) and in vivo (the blood/urine drug concentration method). This review introduces and compares these eight methods in detail.

## 2. Developments of in Vitro Methods

In vitro methods are simple and convenient to perform. It is easy to control the experimental conditions and environment, and the results are reproducible. However, these methods cannot fully reflect the actual absorption of nanoparticles in vivo, and are therefore usually used to study intestinal absorption mechanisms. In vitro methods for the study of the intestinal absorption of nanoparticles include the dialysis bag, rat gut sacs, the Ussing chamber, and cell culture models (e.g., Caco-2 monolayer, and co-cultures of Caco-2 and HT29-MTX).

### 2.1. Dialysis Bag

**Figure 1:**
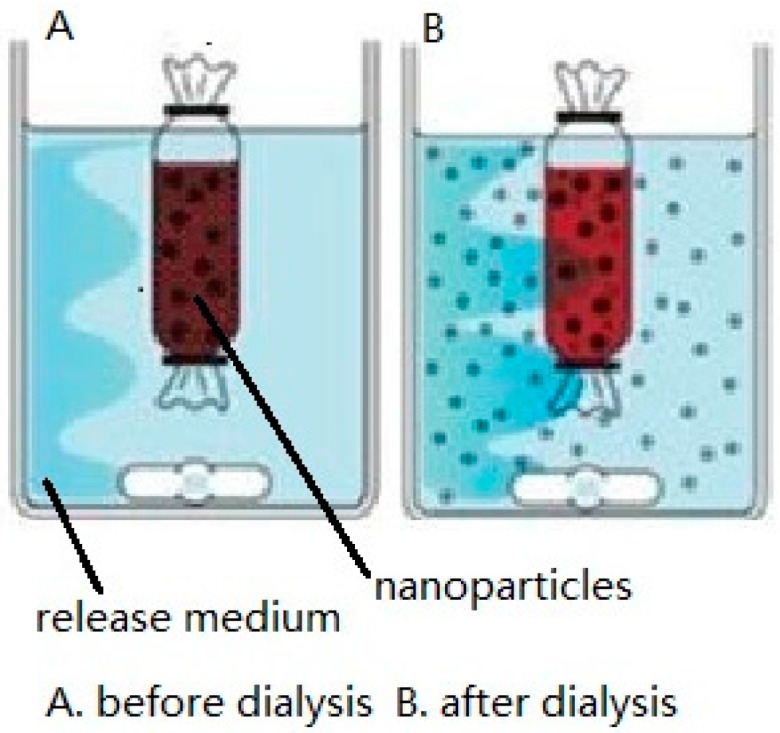
Positive dialysis method (**A**,**B**).

**Figure 2:**
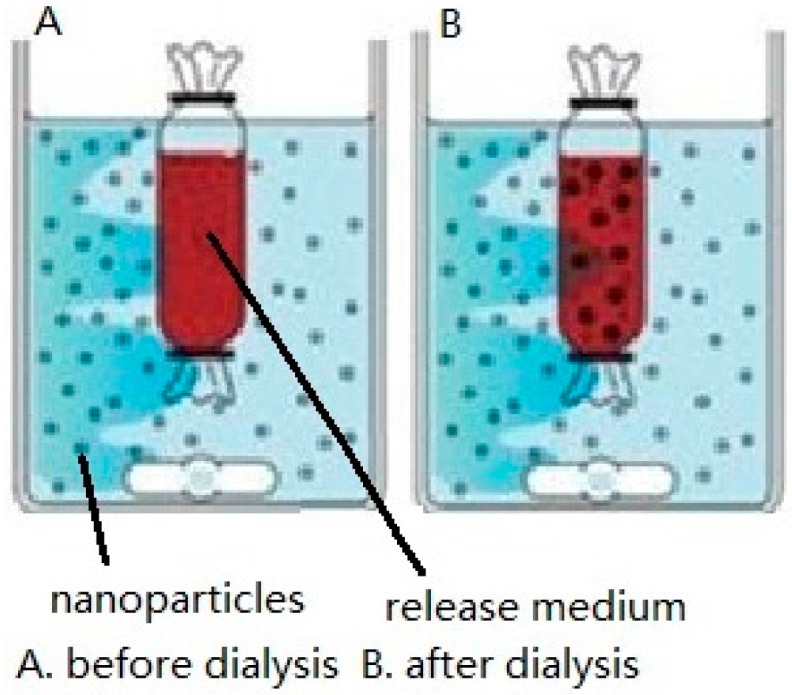
Reverse dialysis method (**A**,**B**).

The dialysis bag method [14,15] includes the positive dialysis method and the reverse dialysis method as shown in Figure 1 and Figure 2. It can be used to simulate drug release in vitro, as dialysis bags can intercept substances of a specific molecular weight. Ideally, drug diffusion through the dialysis membrane should be maintained at the highest rate possible to prevent the dialysis membrane from affecting drug release. The pore size of the dialysis membrane (i.e., the molecular weight cut-off, MWCO) must therefore be specifically selected to ensure free diffusion of the drug. The release medium external to the dialysis bag consists of artificial gastrointestinal fluid at the appropriate pH that may also contain an appropriate surfactant to enhance drug solubility [16]. In addition, the sink condition must also be considered based on the choice of the release medium and drug content. The sink condition refers that the concentration of drugs in the release medium is far less than its saturation concentration. In physiology, it refers that drugs are absorbed quickly in vivo. Generally, the volume of the release medium for drug release test is 3–5 times that of the saturated solution volume.

As shown in Figure 1, in the positive dialysis method, the NP solution is placed in the dialysis bag, which, in turn, is placed in the artificial intestinal fluid [17]. The drug is slowly released into the outside release medium from the dialysis bag due to osmotic pressure arising from the concentration difference. Samples are collected from the outside release medium at regular time intervals, with the same volume of fresh release medium being added directly afterwards. The drug release kinetics is obtained by measuring the accumulative release of the drug [18,19]. This method enables easy sampling of the outside release medium and avoids the loss of NPs during the sampling. However, the NP solution suffers reduced mixing with the release medium, as robust stirring is difficult to achieve in the dialysis bag [20].

As shown in Figure 2, in the reverse dialysis method, the dialysis bag is filled with artificial intestinal fluid and the NP solution is placed in the external release medium. The NP-released drug will slowly enter the dialysis bag due to the concentration difference. Samples are taken from within the dialysis bag at predetermined time intervals to measure the accumulative drug release from the NPs, with the same volume of fresh release medium being added directly afterwards [21,22]. Compared with the conventional positive dialysis method, reverse dialysis significantly reduces dialysis rate variability because the NPs mix better with the release medium and simulates the in vivo environment better. However, it is more difficult to sample from inside the dialysis bag compared with the positive dialysis method.

Tariq et al. [18] prepared epirubicin (EPI)-NPs and performed a release study of EPI using the positive dialysis method. In their study, 2 mg of free EPI and EPI–NPs equivalent to 2 mg EPI were dispersed in 5 mL of dissolution media and then poured into a dialysis bag (MWCO 8–10 kDa) and subsequently immersed in 50 mL of dissolution media. An in vitro release study was carried out in an incubator shaker under mild stirring (100 rpm) at 37 ± 0.5 °C. The dissolution studies were carried out in simulated gastric fluid (pH 1.2) for the first 2 h and simulated intestinal fluid (pH 6.5) thereafter for a further 48 h. Samples were removed at scheduled time intervals (0.25, 0.5, 1, 2, 3, 4, 6, 8, 12, 24 and 48 h) and analyzed with high-performance liquid chromatography (HPLC). The results demonstrated an accumulative percentage release of EPI of (96.23 ± 3)% from the free EPI solution in the first hour, while an accumulative release of (80.94 ± 6)% was observed from EPI–NPs in the first 48 h. The EPI–NPs showed a biphasic release profile, namely an initial burst release followed by a sustained release. Sustained release behavior of the EPI–NPs is favorable because it facilitates a therapeutic effect over an extended period of time.

Our group studied the release characteristics of freeze-dried ursolic acid (UA) self-microemulsion in vitro with the reverse dialysis method. PBS (pH 7.4) containing 1% sodium dodecyl sulfate (an emulsifier) was used as the release medium. Ten dialysis bags (MWCO 8–14 kDa) containing 1 mL release medium were put into the outside release medium and incubated for 12 h at 37 °C. Then, 5.3 g UA self-microemulsion was mixed into the release medium with stirring. One dialysis bag was taken out at 10 min, 20 min, 30 min, 1 h, 2 h, 4 h, 6 h, 8 h, 12 h and 24 h and the drug content (C_i_) in each bag was measured. C_24_ was regarded as the total drug content after complete release of UA from the self-microemulsion and the accumulative release percentage was determined relative to this value. Free ursolic acid solution was similarly tested, and the results showed that the ursolic acid self-microemulsion had significantly sustained release compared with free UA.

### 2.2. Rat Gut Sac

The rat gut sac method includes the everted gut sac method and the non-everted gut sac method. The everted gut sac method has been widely used since Wilson and Wiseman [23] described the use of this method to study intestinal absorption. First, the anaesthetized rat’s abdominal cavity is opened and the small intestine is removed. Next, the intestine is placed over a glass rod and everted, and subsequently placed in Krebs–Ringer solution containing the drug-bound NPs. Samples are taken from both sides of the intestine at fixed time intervals to determine the drug diffusion rate. This process is usually quick considering the loss of tissue activity. The non-everted gut sac method [18] involves placing the NP solution in the rat gut sac without everting first, and intestinal absorption simulated by measuring the decreasing drug content within the intestine.

In previous studies applying the everted gut sac method, healthy adult male Sprague–Dawley (SD) rats were euthanized after fasting for 12 h. A 10-cm portion of the ileum was removed and placed into pH 7.4 artificial intestinal fluid with continuously bubbled 95% O_2_ to provide oxygen and 5% CO_2_ to maintain the pH. Residual blood clots were removed by washing with artificial intestinal fluid and blotting of the ileum segment with clean filter paper. The ileum was then carefully everted using a glass rod, washed again to remove any adherents, and tied in one end with cotton thread. The other end was tied with the proper glass rod. The ileum sac was filled with 37 °C artificial intestinal fluid by syringe. NPs solution was placed in the everted gut sac and saturated with mixed gas consisting of 95% O_2_ and 5% CO_2_. Samples were withdrawn at regular time intervals from the ileum sac to measure the change in drug concentration over time, with the same volume of fresh medium being added back directly afterwards [24].

De Souza et al. [25] developed praziquantel (PZQ)-loaded solid lipid nanoparticles (SLN) and explored the biological application of the rat gut sac method in the intestinal permeation of PZQ. The everted gut sacs were placed in 20 mL TC-199 buffer containing either 250 μg·mL^−1^ of PZQ or an aliquot of PZQ-SLN carrying an equivalent quantity of PZQ. TC-199 buffer is used for tissue culture and is composed of NaCl, KCl, CaCl_2_, Na_2_HPO_4_ and glucose. After 60 min incubation, the sacs were removed and washed with TC-199 buffer, followed by filtration of the drug content present in the interior of the everted rat small intestine segments through a cellulose acetate membrane (0.22 μm pore size). The total PZQ content in each segment was then determined with HPLC. The results showed that the accumulative permeation profile of free PZQ transported across the duodenal segments (22.57% ± 3%) was significantly higher than that of SLN-bound PZQ (11.97% ± 6%). The decrease in intestinal permeation observed for PZQ loaded in the SLN may be attributed to reduced availability of the drug in the mucosal bulk due to its incorporation into the SLN, suggesting that the SLN matrix acted as a reservoir system leading to slower drug diffusion. Thus, PZQ administered with NPs may prove more effective than free PZQ against parasites located in the mesenteric veins of the intestine.

Tariq et al. [18] adopted the non-everted intestinal sac method to assess the permeability potential of epirubicin (EPI)-PLGA-nanoparticles. Non-everted intestinal segments filled with either free EPI solution (1 mL, 100 g·mL^−1^) or EPI–NP suspension (equivalent to 100 g of free EPI in 1 mL) were incubated in 10 mL pre-warmed (37 ± 0.5 °C) and pre-oxygenated Tyrode’s buffer. Samples (0.5 mL each) were collected at 15, 30, 45, 60, 75 and 90 min and replenished with the same volume of fresh Tyrode’s buffer solution. All samples were subsequently analyzed with HPLC. The apparent permeability coefficient (*P_app_*) was calculated with the following equation and expressed in cm·s^−1^ [26]:

(1) $$P_{app}=\;dQ\;/\left(dt\;\times \;A\;\times C_{o}\right)$$

where *dQ*/*dt* was the rate of drug diffusion towards the basolateral side, *C_0_* was the initial concentration over the apical side and A was the surface area of the intestinal tissue (cm^2^). Higher permeation of free EPI compared with NP-bound EPI was observed at each time point and the Papp for the EPI–NPs was found to be 2.78 × 10^−6^ cm·s^−1^, which was significantly higher (*p* < 0.0001, ~4.49-fold) compared with free EPI (0.619 × 10^−6^ cm·s^−1^). Hence, it is envisaged that PLGA-NPs (poly(lactic-co-glycolic acid)-NPs) may be a prospective platform for effective oral delivery of epirubicin.

The non-everted and everted rat intestinal sac methods are widely used in in vitro absorption models to assess transport mechanisms and to predict in vivo absorption of drugs in humans [26,27,28,29]. The non-everted sac model has several advantages over the everted sac model, such as greater simplicity, lower sample volume requirements, and amenability towards successive collection of serosal samples with less intestinal morphological damage as a result of the absence of eversion [30]. The most common disadvantage of the everted gut sac model is morphological damage to intestinal tissue while everting [31].

### 2.3. Ussing Chamber

**Figure 3:**
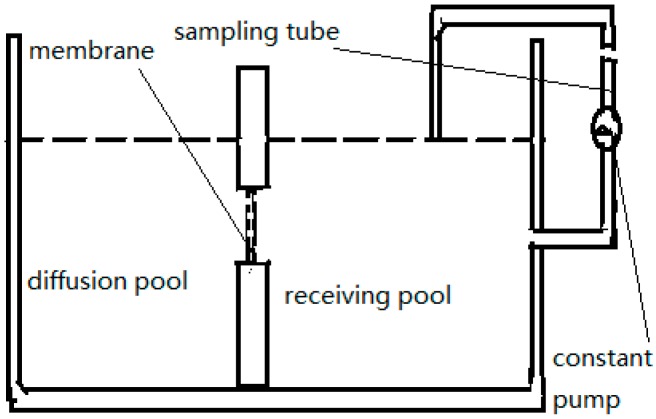
The Ussing chamber.

This method was developed by Ussing [32] and his colleagues when studying the active transport of sodium as the source of electric current in the short-circuited isolated frog skin. The Ussing chamber [10,33] as shown in Figure 3, is an instrument in which human or animal intestines or mucous membranes are fixed between a receiving pool and a diffusion pool containing the NPs. After a period of incubation, the drug concentrations on both sides of the membrane are measured to determine the rate of drug absorption from serosa to mucosa.

In this method, an appropriately sized intestinal segment is taken from the rat’s abdominal cavity. After cleansing with artificial intestinal fluid, the segment is fixed on a glass rod. The dermal layer of the tissue is scraped carefully with a scalpel to expose the active isolated intestinal mucosa. The active mucosa is then fixed in the Ussing chamber in a 37 °C, mixed-gas environment consisting of 95% O_2_ and 5% CO_2_ [34]. At fixed time intervals, samples are collected from the receiving pool and replaced with the same volume of fresh medium pre-equilibrated to 37 °C. Samples are removed at different time intervals and analyzed to obtain the drug concentration, thereby allowing analysis of the intestinal absorption of drugs from the NPs. The *P_app_* representing permeability of the test compound from the mucosa to serosa is calculated using the following equation and expressed in cm·s^−1^ [35,36,37,38,39]:

(2) $$P_{app}=\;dQ\;/\left(dt\;\times \;A\;\times C_{o}\right)$$

where *dQ*/*dt* represents the flux of the drug from the diffusion pool to the receiving pool, *C*_0_ is the initial concentration of the drug in the diffusion pool and A is the area of the intestinal membrane used.

Rekha et al. [40] adopted this method to evaluate the permeation-enhancing capacity of thiomalyl chitosan (TCS) particles. Native chitosan (NC) or thiomalyl chitosan (TCS) particles (500 µL each) in phosphate buffer (10 mg·mL^−1^) was applied onto the intestinal patch, and fluorescein dextran (FD4) was used to evaluate permeation. Samples were collected at 1 h intervals for 3 h and the amount of permeated FD4 was determined with fluorescence spectroscopy. The *P_app_* was then calculated. The TCS particles were observed to increase the permeability of FD4 across the intestinal tissue. After 3 h, 9.12 ± 0.03 μg of TCS-bound FD4 had been permeated, while only 5.67 ± 0.02 μg of NC-bound FD4 had been permeated. The apparent permeability of FD4 using NC and TCS particle-based delivery was 3.50 × 10^−3^ cm·s^−1^ and 5.75 × 10^−3^ cm·s^−1^, respectively, while the *P_app_* for free FD4 was 4.4 × 10^−4^ cm·s^−1^. The enhancement ratio was therefore 7.95 and 13.07 for the NC and TCS particles, respectively. The conclusion was that TCS particle-based delivery may significantly improve permeation across the intestine.

The Ussing chamber is suitable for studying the intestinal absorption of NPs because of the following characteristics: the activity of the small intestine can be estimated by determining the resistance of the intestinal membrane, the absorption of different segments of intestine can be studied, and the test samples are clean and easy to be analyzed. Nonetheless, this method has disadvantages such as a lack of blood and nerve supply, which results in vulnerability of the mucosa [10], rapid loss of mucosa activity, and relatively low-transport [37] during the procedure. 

### 2.4. Cell Culture Model

To study the mechanisms of drug absorption at the cellular and molecular levels, many different cell models have emerged such as Caco-2, HT29-MTX, and so forth. The Caco-2 cell model is used as an in vitro model of intestinal epidermal cellular drug transport and metabolism, and has been widely used in the screening of orally administered drugs and in research on the drug intestinal absorption process. It has been shown to be an appropriate model for studying drug absorption.

#### 2.4.1. Caco-2 Monolayer

**Figure 4:**
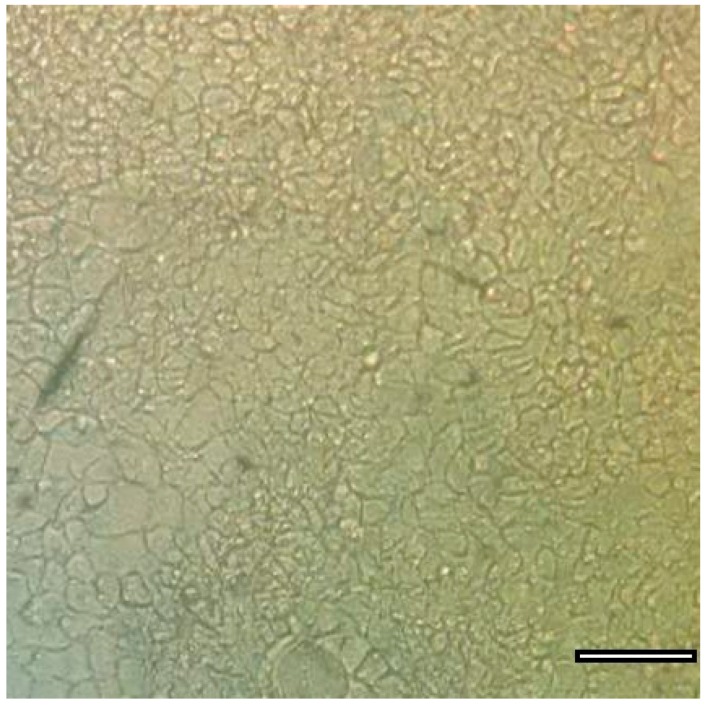
Caco-2 cells used for drug studies (scale bar = 100 μm).

**Figure 5:**
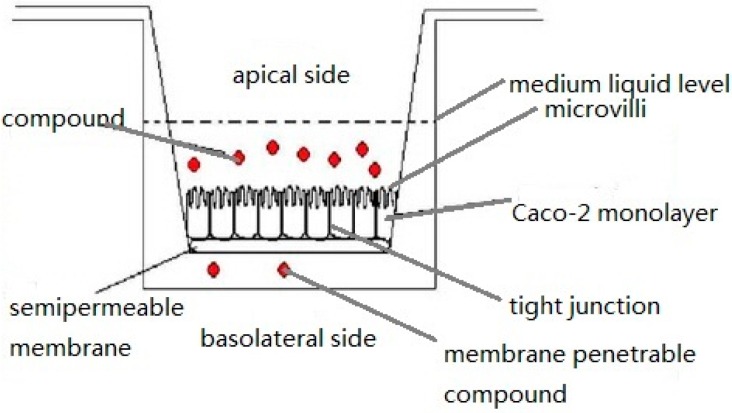
The Caco-2 cell permeability method.

The human adenocarcinoma cell line Caco-2, shown in Figure 4 [41], has been developed as a model for studying intestinal epithelium cellular drug absorption, transport and metabolism [37,42] and was first proposed by Hidalgo et al. in 1989 [43]. Caco-2 cells were originally isolated from human colon adenocarcinoma by Fogh et al. [44]. The Caco-2 cell monolayer is similar to the small intestinal epithelial layer based on differentiation into columnar absorptive cells, including tight junctions and brush borders, and the presence of similar enzymes and carrier-mediated transport systems [9,14]. It is a widely accepted in vitro model for predicting intestinal absorption by epithelial cells [45,46], and this model also exhibits enterocyte-like characteristics [47]. For transcellular transport assays, Caco-2 cells are grown in transwell plates as shown in Figure 5 in DMEM-based culture medium (dulbecco’s modified eagle medium) for 18–21 days until a constant transepithelial electrical resistance (TEER) is achieved, indicating that tight junctions have formed in the monolayer [17]. Before the initiation of permeation experiments, the cell membranes are equilibrated in transport buffer. Hank’s Balanced Salt Solution (HBSS, generally used for the preparation of cell culture media or washing cells) of different formulations and simulated intestinal fluids (SIFs) are added to the apical part of the transwell as the transport buffer, and in all wells, HBSS buffered with 10 mM HEPES (*N*-2-Hydroxyethylpiperazine-*N*-2-ethanesulfonic acid, a hydrogen ion buffer used to adjust pH, pH 7.4) is added to the basal chamber. After pre-incubation with transport buffer, the TEER of the monolayer in each well is measured as the initial value, and permeation studies are initiated by replacing transport buffer in the apical chamber of the transwell with the drug dissolved in the same transport buffer [48,49]. The drug concentration changes over time are analyzed with HPLC and used to calculate the P_app_ using the following equation and expressed in cm·s^−1^:

(3) $$P_{app}=\;\left(dc\;\times \;v\right)/\left(dt\;\times \;A\;\times C_{0}\right)$$

where *dc*/*dt* is the flux across the monolayer (mM·s^−1^), V is the volume in the receiver chamber (mL), A is the surface area of the monolayer (cm^2^), and *C*_0_ is the initial concentration (mM) in the donor compartment. The flux across the monolayer describes the amount of drug transported over time and is calculated from the slope of the regression line obtained from the linear part of the curve [50]. The drug transport mechanism can be calculated using *P_app_* of the study drug at different concentrations.

Lin Qing et al. [51] used Caco-2 cells to examine the uptake and transport of imperialine in vitro. The results showed that in Caco-2 cells, the uptake of imperialine increased with increasing pH of the medium but was unaffected by temperature. The apparent absorptive and secretive coefficients were (8.39 ± 0.12) × 10^−6^ cm·s^−1^ and (7.78 ± 0.09) × 10^−6^ cm·s^−1^, respectively. Furthermore, neither the P-glycoprotein inhibitor verapamil nor the Niemann–Pick C1-Like 1 transporter inhibitor ezetimibe affected the absorption and secretion of imperialine in vitro.

Liu et al. [52] used Caco-2 cells to prove that oligoarginine-modified biodegradable NPs could improve the intestinal absorption of insulin. Caco-2 cells were grown in 24-well transwell culture plates in DMEM medium for three weeks [53]. Coumarin-6-NP (C6-NP), l-poly(arginine)_8_-coumarin-6-NP (l-R8-C6-NP) or d-poly(arginine)_8_-coumarin-6-NP (d-R8-C6-NP) were dispersed in 0.4 mL HBSS on the apical side, and 0.6 mL HBSS was added to the basal chambers. The cells were incubated at 37 °C with the NPs and 0.2 mL samples were collected from the basal medium at predetermined time intervals, with fresh HBSS of the same volume being added directly afterwards. The coumarin-6 concentration of the samples was then measured with fluorescence spectrophotometry. The results showed that the cellular transportation percentage of 3% R8-modified nanoparticles was 2.95-fold (l-R8-C6-NP, *p* < 0.01 vs. C6-NP) or 3.23-fold (d-R8-C6-NP, *p* < 0.01 vs. C6-NP) higher at 4 h than that for the unmodified NPs at the lower concentration tested, and 1.31-fold (l-R8-C6-NP, *p* < 0.01 vs. C6-NP) or 1.89-fold (d-R8-C6-NP, *p* < 0.01 vs. C6-NP) higher at the higher concentration. The R8 modification therefore resulted in a significant improvement of transportation across the Caco-2 cell monolayer at higher concentrations of coumarin-6. The difference between l-R8 and d-R8 in terms of effect on drug transportation therefore became evident when testing increasing concentrations of NPs [52].

The Caco-2 cell monolayer has become one of the most important classic models used to study the intestinal absorption of NPs in vitro. Since the Caco-2 cell line is derived from human colon adenocarcinoma, it provides a good model for simulation purposes. It can be used to distinguish different absorption pathways in the intestinal cavity and to determine the mechanisms and kinetics of drug absorption [54,55]. It is an ideal in vitro model for the study of drug absorption and is suitable for the early stages of drug development. However, one obvious disadvantage of the Caco-2 monolayer is its impermeability to hydrophilic or paracellular transport, which makes it a better model for simulating colonic tissue and restricts its reliability as a model of small intestinal tissue [13,56]. The Caco-2 monolayer has other drawbacks, such as the lack of the mucous layer found in the intestinal wall and a lack of some of the intestinal metabolic enzymes, as it only models epithelial cells in the intestinal epithelium while many other cell types are present in the intestine including mucosal cells and M cells. Co-cultures are therefore an important addition to the Caco-2 monolayer culture and these are used to help better simulate the multicellular intestinal epithelium [13].

#### 2.4.2. Co-Cultures of Caco-2 and HT29-MTX

As mentioned above, the Caco-2 cell model differs from enterocytes and the in vivo physiological environment in various ways. Several modifications and improvements of this model need to be investigated to generate a more predictable cell model. However, co-cultures of Caco-2 cells and e.g., mucus-producing goblet cells can provide a drug absorption model that incorporates the mucus barrier to drug absorption. The addition of mucin-secreting goblet cells in cell cultures modulates the tight junction geometry and yields a mucus gel that covers the whole cell surface as an additional transport barrier. Therefore, co-cultures of Caco-2 cells with goblet and other mucus-secreting cell lines such as HT29-MTX have been proposed in order to solve the issues faced when using Caco-2 cell monolayers alone [37,42]. HT29-MTX is a goblet cell clone with a large proportion of mature goblet cells, established from the human intestinal cell line HT29 [50]. HT29-MTX is used to help simulate and examine the effect of a mucus gel layer in co-culture with cell monolayer models, by contributing mucus-secreting properties [57].

For permeability studies, a mixture of Caco-2 and HT29-MTX cells are seeded on semi-permeable polycarbonate Transwell filter inserts (Costar Transwell, Millipore Corp., Bedford, MA, USA) [8,37,58]. Predetermined cell numbers of Caco-2 and HT29-MTX cells are mixed prior to seeding to yield a specific ratio of Caco-2 to HT29-MTX cells. Co-cultures are maintained under the identical conditions used for stem cultures of Caco-2 cells alone. After 18–22 days in culture, the TEER of the monolayers is measured to estimate cell integrity. The permeability study and calculation methods used are the same as for the Caco-2 monolayer.

Hong Yuan et al. [59] studied the potential of PEGylated solid lipid NPs (pSLN) as mucus-penetrating particles (MPP) for oral delivery across the gastrointestinal mucus. In their study, Caco-2 and HT29-MTX cells were resuspended at ratios of 100:0, 90:10, 75:25, 50:50 and 0:100, seeded at a density of 6.0 × 10^5^ cells.cm^2^ and cultured on polycarbonate filter membranes with a pore size of 0.4 μm and a surface area of 1.12 cm^2^. Compared with unPEGylated SLN, the pSLN showed decreased permeability through the Caco-2 cell monolayer, while showing increased permeability through a mucus-secreting Caco-2/HT29-MTX co-culture cell monolayer. This result indicated that the mucus layer can have a significant impact on determining the efficacy of oral nanoformulations. The *P_app_* of doxorubicin (DOX) through the Caco-2/HT29-MTX (75:25 ratio) cell monolayer was increased by five-fold when delivered by pSLN-10%, while an increase of 3.9-fold was observed for delivery by SLN. The relative bioavailability of SLN was significantly improved by modification with PEG(polyethylene glycol), suggesting that PEG is a potent mucus-penetrating agent and may therefore prove to be a promising nanoparticle modification for oral drug delivery.

## 3. Developments of in Situ Methods

In situ methods mainly refer to experiments on whole animals, where complete blood supply and nerve domination are present, and most importantly, where the intestinal nerve remains intact. These methods directly reflect drug absorption in situ, and are therefore commonly used to study drug penetration and absorption kinetics. In situ methods include intestinal perfusion, intestinal loops, and intestinal vascular cannulation.

### 3.1. Intestinal Perfusion

**Figure 6:**
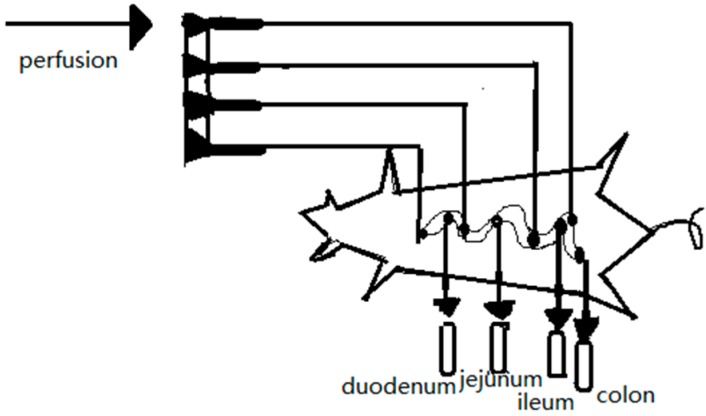
Schematic diagram of intestinal perfusion.

This method was first proposed by Curran et al. [60] for studying ion and water fluxes in the ileum of rats. Previous studies have demonstrated that rat and human jejunum effective permeability (*P_eff_*) estimates of passively absorbed solutes exhibit high correlation, and both can be used with precision to predict in vivo oral absorption in humans. Studies of carrier-mediated transport requires scaling between the two model species, since the transport maximums and/or substrate specificities may differ [61]. The general experimental procedure is as follows. First, the perfusion tube and drainage tube are inserted by laparotomy into the proximal and distal intestinal segments, respectively. Second, the drug solution is poured into the intestinal cavity with a peristaltic pump at a specific rate and the effluent is collected as shown in Figure 6. The drug and tracer concentrations are determined and used to calculate the drug absorption rate and *P_eff_*, respectively. Based on the different patterns of perfusion used, intestinal perfusion is divided into circular perfusion and single-pass perfusion.

#### 3.1.1. Circular Perfusion Method

In this method, the perfusion fluid containing NPs is poured into the intestine through a peristaltic pump and returned back to the container from the other end of the intestine at a rate of 2.5 mL·min^−1^. The drug and tracer concentrations in the perfusion fluid are measured before and after perfusion and used to calculate the absorption constant. These experiments typically require two or more hours to complete, during which time the animals must be kept stable.

**Figure 7:**
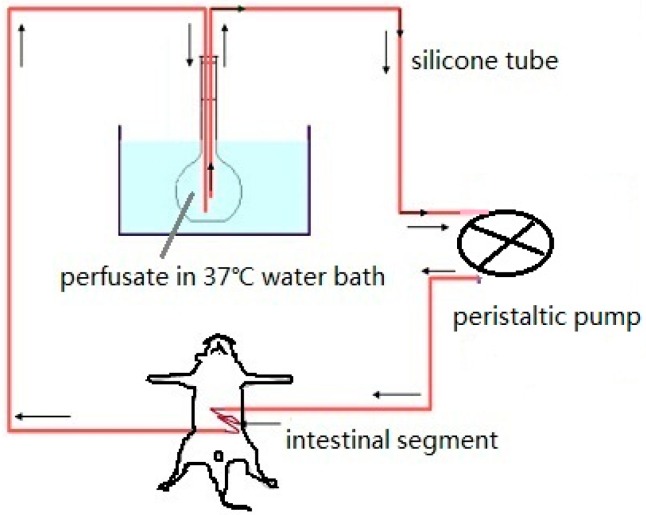
Circular perfusion.

The specific experimental details of this method are as follows. Rats fast for 24 h beforehand are anesthetized and restrained in a supine position. The intestinal segments are exposed by making a 3 cm incision along the abdominal cavity through the ventral midline, and two medical silicone tubes are cannulated at both ends of the intestine. The upper tube is connected with the peristaltic pump to import the perfusion fluid, while the lower tube is used to export the perfusion fluid as shown in Figure 7 [62]. The incision is then covered with absorbent cotton saturated with physiological saline and warmed with infrared light. The intestinal contents are washed out with physiological saline warmed to 37 °C. The residual physiological saline in the intestine is flushed out with air, following by the NP solution containing phenol red being added into the intestinal circulation [52]. Samples are taken at fixed time intervals, with an equivalent volume of Krebs–Ringer solution added back into the perfusion directly afterwards. Perfusate samples are centrifuged and divided into two aliquots. One aliquot is used to determine the drug concentration rate, and the second aliquot is used to determine the *P_eff_* based on the phenol red levels determined with a UV-based assay. At the end of the study, the perfused intestinal segment is excised, drained and the length is measured [63]. By plotting the drug-release kinetic curve researchers can calculate the release kinetics thereby obtaining the absorption rate constant (*k*_a_) and the permeability coefficient (*P*, expressed as distance per unit time, has been commonly interpreted to represent the velocity of drug movement across a heterogeneous medium such as skin and intestinal epithelium [64]).

Our group previously studied the intestinal absorption kinetics of gastrodin, acyclovir, ferulic acid and paeoniflorin using the circular perfusion method in rats. The results showed that gastrodin was well absorbed in the upper and central sections of the small intestine by passive diffusion. Acyclovir was mostly and poorly absorbed by the upper and middle intestinal tract. The absorption of ferulic acid and paeoniflorin in rat intestine was determined to be a first-order process with a passive diffusion mechanism. Ferulic acid and paeoniflorin was absorbed by whole intestinal segments, and sustained-release formulations of these two compounds have since been prepared.

Sun et al. [65] introduced a new oral delivery system, polybutylcyanoacrylate NPs (PBCNs), to improve the oral bioavailability of curcumin (CUR) and to study the effect of the NPs on intestinal absorption of CUR. Using the circular perfusion method, the drug permeability of various segments of the intestine, including the duodenum, jejunum, ileum and colon, was determined to test whether intestinal absorption of PBCN-encapsulated CUR exhibited any intestinal site-dependent properties. There was a significant difference in absorption observed among the different intestinal segments (*p* < 0.05). While CUR encapsulated in the PBCNs could be absorbed by all intestinal segments tested, the main absorption segments were the ileum and colon.

The in situ circular perfusion method is widely used for studying drug intestinal absorption behavior particularly in the early stages of drug development [66]. The blood vessels and nerves do not need to be cut off during these experiments, which means that the perfusion intestinal segment remains intact, thus better simulating the in vivo environment. In addition, this method involves a simple operation. However, the addition of phenol red into the perfusate used to adjust the influence of water absorption may also be absorbed, and this may affect the intestinal transport of some compounds and their analysis in rats. Furthermore, NPs-bound drugs must be dissolved in the perfusate.

#### 3.1.2. Single-Pass Perfusion

The difference between single-pass perfusion and circular perfusion is that, in the former, the perfusate does not return to the original drug container but to another, end-point collection container. In single-pass perfusion, the perfusate is collected from the drainage tube and used to determine the decrease in drug concentration over time, thus allowing calculation of the pharmacokinetic parameters.

**Figure 8:**
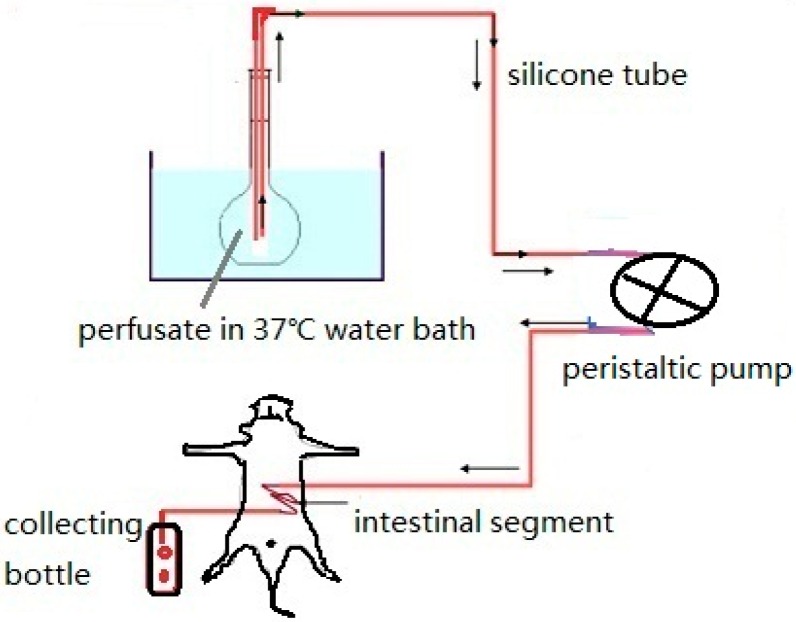
Single pass perfusion.

The experimental animals are treated the same as in the circular perfusion method, except that the tubes from the proximal and distal intestine are connected with a weighted bottle as shown in Figure 8. The test NP solution is poured into the intestinal segments at a flow rate of 2 mL·min^−1^. When the solution appears at the end of the intestinal segment, the flow rate is reduced to 0.2 mL·min^−1^ [24]. This flow rate is maintained for 105 min over which time the perfusate is collected. The drug supply bottle and collecting bottle are replaced every 15 min. Based on changes observed in the bottle weight and drug content, drug release kinetic curves can be generated and used to calculate the effective permeability coefficient of the study drug [67,68,69].

Parvin et al. [70] studied the permeability of clarithromycin (CLA)-PLGA NPs using the single-pass intestinal perfusion technique in rats. Permeability coefficients (*P_eff_*) in anaesthetized rats were determined at three different concentrations of free and NP-bound CLA. Free drug solution or NP suspensions in phosphate-buffered saline (PBS) were perfused through a cannulated jejunal segment and samples were taken from the outlet tubing at different time points up to 90 min. The mean *P_eff_* for the free CLA solution at concentrations of 150, 250 and 400 μg·mL^−1^ were found to be (1.20 ± 0.32) × 10^−3^, (9.62 ± 0.46) × 10^−4^, and (1.36 ± 0.95) × 10^−3^ cm·s^−1^, respectively. The corresponding values for the same concentrations of NP-bound CLA were found to be (2.74 ± 0.73) × 10^−3^, (2.45 ± 0.88) × 10^−3^, and (3.68 ± 0.46) × 10^−3^ cm·s^−1^, respectively. This study showed that the intestinal permeability of CLA-bound NP suspensions was significantly increased in comparison with the free drug in solution.

Jin Xin et al. [71] studied the oral absorption of a nanostructured liquid crystalline formulation of 20(*S*)-protopanaxadiol (PPD) using the single-pass intestinal perfusion technique in rats. Four segments of the intestine (duodenum, upper jejunum, terminal ileum and colon) were perfused simultaneously with a perfusate containing 30 μM PPD or PPD-loaded cubic NPs. When using the PPD-cubosome, the *P_eff_* of PPD was increased significantly across all four areas of the rat intestine (*p* < 0.05) compared with free PPD in solution. Hence, the cubic nanoparticles may prove a promising oral carrier for PPD.

This method requires a simple operation and is readily reproducible. It provides a good simulation of the in vivo environment because it is not sensitive to pH variations as a result of the preservation of the microenvironment above the epithelial cells, and it provides intact blood supply to the intestine throughout the experiment [70]. In addition, other experimental conditions such as NP concentration and intestinal perfusion rate are controllable and repeatable. However, this method involves measurement of the volumes of intestinal perfusion by weight, thus resulting in some experimental deviation [72]. The absorption rates obtained with this method using the rat model are reproducible and exhibit good correlation with results obtained with human intestine [61].

### 3.2. Intestinal Loop Method

This method was first introduced by Kannikar in 1971 [73]. Rats are first anaesthetized, the abdomen is incised open, and the desired segment of intestine is washed, ligated and then injected with a specific concentration of NPs into the intestinal loop. The whole gastrointestinal tract is carefully replaced into the abdominal cavity, and the incision is closed with clamps and kept moist by covering it with gauze pads presoaked in normal saline. After no more than 24 h, the rat is euthanized and the intestines are removed for analysis [13]. After performing a closed-loop experiment, the tissue is typically flash frozen and the fluorescence or morphology of the intestine is determined with microscopy [74]. For a more quantitative result, the frozen tissue can be dissolved in acetonitrile and then evaluated for drug content, or the remaining NP solution can be collected to measure the residual drug content, thus allowing calculation of the absorption of NPs by the intestinal segments [59,74,75].

Yuan Hong et al. [59] applied the ligated intestinal loop model to evaluate the potential of PEGylated solid lipid NPs (pSLN) as mucus-penetrating particles (MPP) for oral delivery across gastrointestinal mucus. SLN/FITC or pSLN-10%/FITC K-R culture solutions (100 μg·mL^−1^, 0.5 mL) with equal fluorescent intensities were injected into the jejunum loop, which was then ligated at both ends. After 2 h, the loop was flash frozen and visualized with inverted two-photon confocal microscopy. The ligated intestinal loop model in vivo demonstrated that pSLN could rapidly penetrate mucus secretions, whereas the SLN were largely trapped by the highly viscoelastic mucus barriers.

Using the intestinal loop method, our group successfully studied the intestinal absorption characteristics of insulin in the presence of absorption enhancers, and determined the sites of increased intestinal insulin absorption. Insulin solution was co-administered with different absorption enhancers to the various intestinal loops of fasted rats, including the ileum, ascending colon, transverse colon, descending colon and the sigmoid colon. The results showed that the ileum was the major site of insulin absorption and that the colon mucosa was more sensitive to the effect of absorption enhancers than the small intestinal mucosa.

It is easier to perform this method compared with in situ intestinal perfusion. However, since the intestine secretes large quantities of digestive fluids during the experiment and there may be a lot of food residue in the lumen, it can prove difficult to analyze the samples. Another disadvantage of this model is the variation between NP uptake in the small intestine of different animals [13]; for instance, one study showed an order of magnitude difference in uptake between the rat intestinal loop model and that of the in vivo murine model [76].

### 3.3. Intestinal Vascular Cannulation

**Figure 9:**
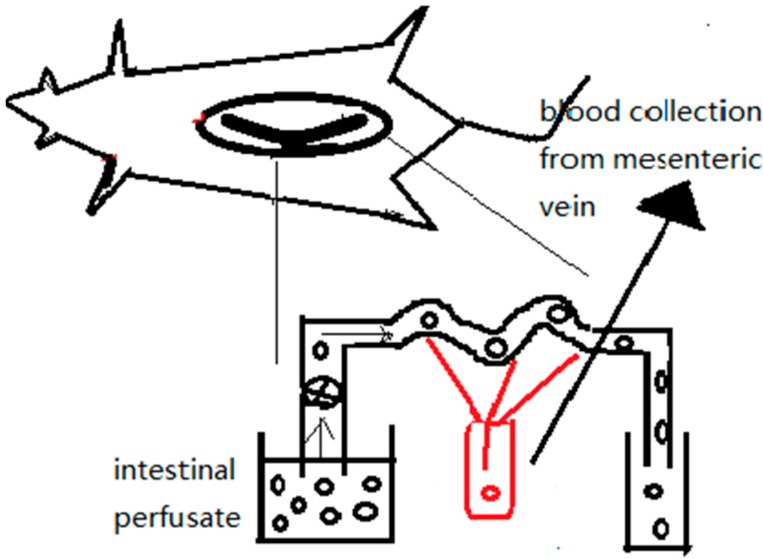
Mesenteric vessel blood sampling in combination with intestinal perfusion.

The in vivo intestinal vascular cannulation method as shown in Figure 9 [77] involves extraction of the mesenteric artery or portal vein blood to obtain the blood-based drug content after oral administration of drug-bound NPs. In this way, researchers can simulate intestinal absorption in vivo to obtain the direct absorption of drugs from the lumen into the circulation. The NP solution is intragastrically administered to healthy male SD rats that have been fasted for 12 h before the experiment. Mesenteric vein cannulation in combination with intestinal perfusion experiments can improve insights into intestinal drug absorption mechanisms.Matsuda et al. [78] assessed the intestinal availability of various drugs following oral administration using portal vein-cannulated rats. Animals were implanted with catheters in the portal vein as follows. After anesthetization and incision of the abdominal cavity, the portal vein of the rat was detached near the liver. To prevent bleeding, the portal vein was ligated temporarily as the catheter was inserted. The catheter was fixed with a purse-string suture on the portal vein. The other end of the catheter was then passed subcutaneously to the dorsal base of the neck.

Usansky et al. [79] used portal vein-cannulated rats to study saquinavir (SQV) absorption under chemical inhibition of P-glycoprotein (Pgp), multidrug resistance-associated protein 2 (MRP2), and/or CYP3A (midazolam). The results provided direct evidence that the low oral absorption of SQV was controlled by the secretory transporter Pgp rather than by limited passive diffusion, owing to its poor physicochemical properties. Pgp-mediated transport was also responsible for the highly variable oral bioavailability of SQV. In contrast, intestinal MRP2 and intestinal CYP3A appeared to play minor roles in SQV oral bioavailability.

Measurement of the absorption of drugs with this method is determined by calculating the amount of drug absorbed into the blood, which is not limited by the size of the experimental animals nor the blood volume used. This method can therefore provide a true reflection of the permeability of drugs across the small intestine [80]. However, drugs are susceptible to the effects of catabolism in the blood circulatory system and trauma occurs in the rat abdomen during the experiment. In addition, the operation in this method is hard to master. All of these challenges limit the widespread application of this method.

## 4. Development of in Vivo Methods

No matter how sophisticated an in vitro model is, in vivo evaluation will eventually be required to validate the true performance of an oral drug delivery system. Noninvasive monitoring is naturally preferred, but such options and the type of data extracted are inherently limited. The most relevant information required includes drug release kinetics and biodistribution of the NPs. The method below describes a commonly used approach for analyzing the intestinal absorption of orally delivered NP-bound drugs [13].

The NP intestinal absorption in vivo method [18,81] requires the collection of blood or urine samples after oral administration of drug-bound NPs at specific time points [82]. Both the whole blood and plasma can be used to perform the assays. The plasma/urine drug concentration is measured with HPLC to obtain the drug release kinetics curve and pharmacokinetic parameters such as C_max_, T_max_ and AUC_0-t_ (C_max_ is the maximum blood/urine drug concentration, T_max_ is the time taken to reach the maximum blood/urine drug concentration, and AUC_0-t_. is the area under the drug concentration vs. time curve), thereby allowing the rate and degree of NP intestinal absorption to be measured [8,38,83,84,85].

Virginia et al. [86] studied the pharmacokinetics of orally administrated paclitaxel bound to PEGylated poly(anhydride) NPs. After oral administration, blood samples of 300 μL each were collected at different time post-administration and then centrifuged for 10 min at 10,000 rpm. The supernatant plasma fraction was stored at −80 °C until HPLC analysis. The results showed that when paclitaxel was loaded on the PEGylated poly(anhydride) NPs, sustained plasma levels of this anticancer drug were observed. In addition, the mean residence time (MRT) of the drug in the plasma and the T_1/2_ were found to be higher when paclitaxel was orally administered as part of an NP formulation than when administered intravenously as Taxol.

Tariq et al. [18] studied the pharmacokinetics of epirubicin (EPI)-bound NPs in rats. Rats were divided into two groups (*n* = 6 each), and each group was treated with an oral dose of EPI of 10 mg·kg^−1^. The first group was treated with free EPI while the second group received an EPI–NP suspension. Blood samples (0.2 mL) were withdrawn at 0.5, 1, 2, 3, 4, 6, 8, 12, 24 and 48 h under mild anesthesia. Drug concentration analysis was carried out with HPLC. The results showed that higher C_max_, T_max_ and AUC_0−t_ values and a lower elimination rate constant were achieved with the EPI–NPs compared with free EPI. Hence, it is envisaged that NPs could be a prospective platform for effective oral delivery of epirubicin.

This method can truly reflect the intestinal absorption of NP-bound drugs in vivo [87,88,89]. However, the results can be affected by physical and physiological factors that may make it difficult to study the absorption mechanism of drugs at the cellular or molecular level, and may not reflect the absorption of the intestinal tract specifically. In addition, there are many factors affecting the test accuracy during the long and complicated operation process required, including the large inter-individual differences of metabolism in vivo. Thus, this approach is not suitable for rapid screening and large-scale evaluation in early drug development [90,91].

## 5. Conclusions

This review provides an overview of the three classical models used to study NP-bound drug intestinal absorption. These models include eight methods in total, which have been described here in detail (in vitro: dialysis bag, rat gut sac, Ussing chamber, cell culture; in situ: intestinal perfusion, intestinal loops, intestinal vascular cannulation; and in vivo: the blood/urine drug concentration method). The advantages and disadvantages of each model were analyzed, and the most appropriate applications for each were suggested. The accuracy and credibility of each method was described based on specific experimental data in the literature, and the deficiencies of each method were thoroughly discussed.

At present, the three categories of absorption model mentioned above are used to simulate the absorption process of NPs from the intestine into the blood circulation. However, with the increasing pace of drug development and the emergence of new oral pharmaceutical dosage forms [92,93], demands for investigation into the intestinal absorption of NPs have become more stringent in recent years. Data from one intestinal absorption model alone cannot be considered to provide a conclusive result; therefore, simultaneous utilization of two or more independent models is favored by most researchers when studying intestinal absorption [24]. Researchers should therefore be familiar with each intestinal absorption model in order to select the most appropriate research methods to study NP intestinal absorption. In addition, researchers should take a dialectical view of the experimental results and draw objective conclusions. Furthermore, with further progress in medical knowledge and technologies, we believe the future will lead to the development of new, improved intestinal absorption models.

**Table 1:** Advantages and disadvantages of the different intestinal absorption models.

Model			Advantages	Disadvantages
In vitro	Dialysis bag	Positive dialysis	It promotes the exchange of substances internal and external dialysis bags a and avoids the loss of NPs during the sampling.	NP solution suffers reduced mixing with the release medium [20].
		Reverse dialysis	NPs mix better with the release medium.	It is difficult to sample.
	Rut gut sac	Everted gut sac	Drugs can contact well with the intestinal mucosa.	There will be morphological damages to intestinal tissue [31].
		Non-everted gut sac	The procedure is simple. There are minor intestinal morphological changes as sampling [31].	Drugs can not contact well with intestinal mucosa.
	Ussing chamber		It is suitable to study the absorption of different intestine segments. Samples are clean and easy to be analysed.	It lacks blood and nerve supply. The mucosa loses activity easily [10,37].
	Cell culture	Caco-2 monolayer	It can be used to distinguish different absorption pathways in the intestinal cavity, to determine the way of drug absorption and the kinetic parameters of drug absorption [54,55].	It lacks mucous layer and some metabolic enzymes. It is impermeable to hydrophilic or paracellular transport [13,56].
		Co-culture of Caco-2 and HT29-MTX	It mimicks well the intestinal cells [37,42].	It’s hard to co-culture.
In situ	Intestinal perfusion	Circular perfusion	It has complete blood supply and nerve domination.	Animals may die during the experiment. Samples are complicated to test.
		Single-pass perfusion	It has complete blood supply and nerve domination.	Animals may die during the experiment. Samples are complicated to test.
	Intestinal loop		It is easier to operate than intestinal perfusion.	It is difficult to analyse the samples.
	Intestinal Vascular cannulation		It simulates well the intestinal absorption of nanoparticles [80].	It is difficult to sample.
In vivo	Blood or urine detection		It can truly reflect the NP intestinal absorption in vivo [87,88,89].	It can not study the absorption mechanism and reflect the partial intestinal absorption [90,91].
